# Treatment with specific and pan-plasma membrane calcium ATPase (PMCA) inhibitors reduces malaria parasite growth in vitro and in vivo

**DOI:** 10.1186/s12936-022-04228-0

**Published:** 2022-06-29

**Authors:** Puji B. S. Asih, Josephine E. Siregar, Farahana K. Dewayanti, Normalita E. Pravitasari, Ismail E. Rozi, Andita F. M. Rizki, Rifqi Risandi, Kevin N. Couper, Delvac Oceandy, Din Syafruddin

**Affiliations:** 1 Eijkman Institute for Molecular Biology, National Research and Innovation Agency, Jakarta, Indonesia; 2 Division of Infection, Immunity & Respiratory Medicine, The University of Manchester, Manchester, UK; 3 Division of Cardiovascular Sciences, The University of Manchester, Manchester, UK; 4 Department of Parasitology, Faculty of Medicine, Hasanuddin University, Makassar, Indonesia

**Keywords:** PMCA4 inhibitors, Antimalarial activity, *Plasmodium falciparum*, *Plasmodium berghei*, *Plasmodium yoelii*, In vitro and in vivo

## Abstract

**Background:**

Rapid emergence of *Plasmodium* resistance to anti-malarial drug mainstays has driven a continual effort to discover novel drugs that target different biochemical pathway (s) during infection. Plasma membrane Calcium + 2 ATPase (PMCA4), a novel plasma membrane protein that regulates Calcium levels in various cells, namely red blood cell (RBC), endothelial cell and platelets, represents a new biochemical pathway that may interfere with susceptibility to malaria and/or severe malaria.

**Methods:**

This study identified several pharmacological inhibitors of PMCA4, namely ATA and Resveratrol, and tested for their anti-malarial activities in vitro and in vivo using the *Plasmodium falciparum* 3D7 strain, the *Plasmodium berghei* ANKA strain, and *Plasmodium yoelii* 17XL strain as model.

**Results:**

In vitro propagation of *P. falciparum* 3D7 strain in the presence of a wide concentration range of the inhibitors revealed that the parasite growth was inhibited in a dose-dependent manner, with IC_50_s at 634 and 0.231 µM, respectively.

**Results:**

The results confirmed that both compounds exhibit moderate to potent anti-malarial activities with the strongest parasite growth inhibition shown by resveratrol at 0.231 µM. In vivo models using *P. berghei* ANKA for experimental cerebral malaria and *P. yoelii* 17XL for the effect on parasite growth, showed that the highest dose of ATA, 30 mg/kg BW, increased survival of the mice. Likewise, resveratrol inhibited the parasite growth following 4 days intraperitoneal injection at the dose of 100 mg/kg BW.

**Conclusion:**

The findings indicate that the PMCA4 of the human host may be a potential target for novel anti-malarials, either as single drug or in combination with the currently available effective anti-malarials.

## Background

Malaria is currently still a major public health problem around the globe. The World Health Organization (WHO) reported that an estimated 229 million cases of malaria occurred worldwide in 2020 [1] compared with 251 million cases in 2010 and 231 million cases in 2017. In malaria endemic country, such as Indonesia, around 5% of the total population reside in hyperendemic areas with annual parasite incidence (API) > 5. 29% live in mesoendemic areas (API between 1 and 5 per 1000), 56.1% live in hypoendemic areas (API < 1 per 1000) and 52% of the population live in malaria-free areas [2].

Strategies to control malaria include early diagnosis and prompt anti-malarial treatment of infected individuals, provision of long-lasting insecticidal net (LLIN) and indoor residual spraying (IRS) to reduce human contact with infected mosquitoes. However, the rapid development of drug resistance in the malarial parasite and insecticide-resistant anopheline mosquitoes have hampered large-scale efforts at malaria control [3], with cases of parasite resistance also reported in countries, such as Indonesia [2]. As many other infectious agents, the malarial parasite often takes advantage of its host’s protein machinery to proliferate and spread. Hence, targeting the host’s pathways involved in disease susceptibility is a possible approach to find novel treatments for malaria. Targeting the host’s molecules may also reduce the development of drug resistance in the parasite, because the modified biochemical events are beyond the parasite itself. Therefore, it is important to study new biochemical pathways both in the parasite and host cells to enable the identification of new therapeutic targets.

Recent genome-wide association studies have demonstrated that a common single nucleotide polymorphism (SNP) of the *ATP2B4* gene encoding a calcium pump called the plasma membrane calcium ATPase 4 (PMCA4) has a very strong association with resistance against severe malaria [4]. PMCA is a family of ATP driven calcium pumps that ejects Ca^2+^ from the cytosol of most cell types [5]. PMCA is encoded by 4 different genes (named PMCA1–4) where the sequence differences are found mainly at the C and N terminal regions [6]. PMCA1 and PMCA4 appear to be the major isoforms that are ubiquitously expressed in most tissues/organs including red blood cells (RBCs) [7]. An expressed quantitative trait loci (eQTL) analysis has revealed that SNPs within the human PMCA4 gene are associated with lower PMCA4 expression and hence alteration of intra-cellular calcium within the RBCs [8]. It is known that a decrease in Ca^2+^ concentration in the parasitophorous vacuole within RBCs might impair parasite reproduction and maturation [8]. Thus, reduction of intra-cellular Ca^2+^ due to alteration of PMCA4 function or expression may affect the development and structure of intra-erythrocytic stages of the parasite [4]. Furthermore, changes in PMCA4 level or function might affect platelets and endothelial cell functions because these cells are also activated by intracellular Ca^2+^ [9, 10]. The activation of platelets and endothelial cells have been well known to have a crucial role in the pathogenesis of severe malaria by enhancing the sequestration and adherence of infected red blood cells within cerebral vasculature. These findings indicate that inhibiting PMCA4 function may be used as a potential alternative for anti-malarial treatment and the mitigation of malaria cerebral complication [8, 11–15].

The present study was aimed at exploring the effects of treatment with PMCA inhibitors on malaria parasite growth in vitro and analysed whether treatment with specific PMCA4 inhibitor aurintricarboxylic acid (ATA) or pan-PMCA1/4 inhibitor resveratrol affected the growth of *Plasmodium falciparum* 3D7 in the RBC culture. This study found that both ATA and resveratrol have an inhibitory activity against *P. falciparum* growth in vitro, with resveratrol displaying strong inhibition at low concentration. The in vivo experiment on the effect of PMCA4 inhibitors on cerebral malaria showed a modest effect on parasite survival, while resveratrol showed significant inhibition of parasite growth at the concentration of 100 mg/kg BW.

## Methods

### Ethics approval

The study was approved by the Eijkman Institute Research Ethic Committee (EIREC).

### PMCA4 pharmacological inhibitors

The PMCA4 specific inhibitor Aurintricarboxylic acid (ATA, C_22_H_14_O_9_) and general PMCA inhibitor Resveratrol (C_14_H_12_O_3_) were used in this study. ATA, purchased from Sigma-Aldrich (Saint Louis, MO, USA), has been identified as a potent specific pharmacological inhibitor of PMCA4 [16] with IC_50_ of 150 nM for PMCA4 inhibition. ATA has only a minor effect on the second isoform of PMCA expressed in the heart, PMCA1 [16]. Resveratrol (trans-Resveratrol, SRT501) was purchased from SelleckChem and was identified as a potent inhibitor of global PMCA family activity [15]. All compounds were dissolved in either double distillate water (ddH_2_O) or dimethyl sulfoxide (DMSO) for both in vitro and in vivo experiments, and further serially diluted into a working concentration using RMPI-1640 media for in vitro. Artemisinin (Sigma Aldrich, St. Louis, Mo, USA) and sulfadoxine were used as positive control on in vitro and in vivo experiment, respectively.

### Parasite cultivation in vitro

The human parasite *Plasmodium falciparum* strain used in this study was chloroquine-sensitive 3D7. Initially, *P. falciparum* 3D7 strain, blood-stage parasites were cultivated in RPMI medium 1640 (Sigma Aldrich, St. Louis, Mo, USA) supplemented with 10% human serum, 0.005% hypoxanthine, 0.21% NaHCO_3_, 0.596% HEPES, 0.25% gentamicin and 3–5% human erythrocytes. The parasites were grown in Type AB^+^ human blood under controlled and placed in 25 mm^3^ flask and incubated in a candle jar at 37 °C as described by Trager and Jensen method [17] but with modification. All strains were synchronized once with d-Sorbitol 5% [18] 2 days before testing. Parasites was most suitable for drug assays when they were 1–2% parasitaemia, and mostly ring stages with no gametocytes.

### Determination of anti-malarial activity of PMCA inhibitors in *P. falciparum *in vitro

When the *P. falciparum* parasite culture reached 1–2% parasitaemia, it was sub-cultured into 96- well plates (180 µl per well) and incubated in the presence of various concentrations of ATA and resveratrol, ranged from 0 to 10^–9^ nM, obtained through tenfold serial dilutions. Non-parasitized erythrocytes were used as a negative control and parasitized erythrocytes without any test PMCA4 inhibitors were used as positive control. The anti-malarial activity of the PMCA inhibitors was compared with the standard drug, artemisinin. After 48-h incubation, thin Giemsa blood smears were made for each well and the parasite levels were determined by manual counting of parasite under light microscope. All experiments were done in duplicate and the 50% inhibitory concentration (IC_50_) of each inhibitor was determined.

### Parasite cultivation in vivo

*Plasmodium berghei* strain ANKA and *Plasmodium yoelii* strain 17XL parasites (from Prof. Laurent Renia, Agency for Science, Technology and Research (A*STAR), Singapore) from cryopreserved stock was thawed and passaged into C57BL/6 strain mouse as donor and monitored until the parasitaemia level between 1 and 3% [16]. Approximately 1 × 10^4^ infected erythrocytes from the donor [19] were inoculated to the experimental C57BL/6 mice.

### Determination of anti-malarial activity of PMCA inhibitors Aurintricarboxyclic Acid (ATA) against *Plasmodium berghei* in experimental cerebral malaria (ECM) model on in vivo

*Plasmodium berghei*-infected mice were treated intraperitoneally with Aurintricarboxylic Acid (ATA) drug in various concentration (30 mg/kg BW, 10 mg/kg BW, 1 mg/kg BW) daily, started from one day before infection until 9 days post infection. In addition, anti-malarial drug sulfadoxine was used as positive control for this experiment and administered 25 mg/kg BW as its therapeutic dosage. The sulfadoxine treatment was done at 6–9 days post infection. A group without any treatment was also used as a comparison in this experiment. The survival rates of all experimental mice were observed as well as the concentration of red blood cells (RBCs) and body weight during the course of infection. The amount of RBC was examined under microscope using Niewbauer-Chamber. All the observation was followed until day 14 post infection.

### Determination of PMCA4 inhibitor activity in vivo

*Plasmodium yoelii* were inoculated intraperitoneally into BALB/c mice and monitored daily until parasitaemia level of 1–2% with Giemsa-stained blood smear [20]. The PMCA4 inhibitor, resveratrol was given ip at various concentration daily for 4 days, generally started at days 5 post inoculation. In addition, anti-malarial drug sulfadoxine was used as positive control for this experiment and administered 25 mg/kg BW as its therapeutic dosage. The IC_50_ of each drug were analyzed based on parasitaemia level following 4-day treatment. The data was statistically calculated using a probit analysis.

## Results

### Anti-malarial activity of PMCA4 inhibitor, aurintricarboxylic acid (ATA), in vitro

**Fig. 1:**
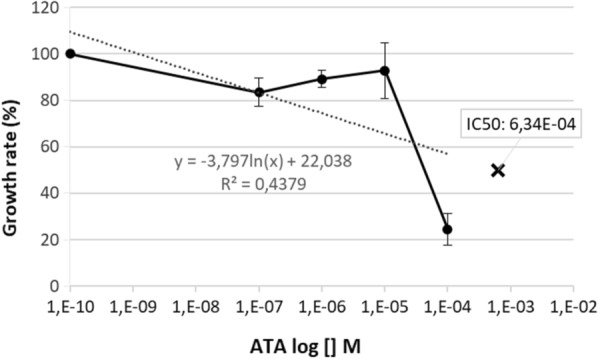
*P. falciparum* 3D7 parasite growth rate after 48 h of incubation with various concentration of ATA. The IC_50_ was detected at 634 μM

**Fig. 2:**
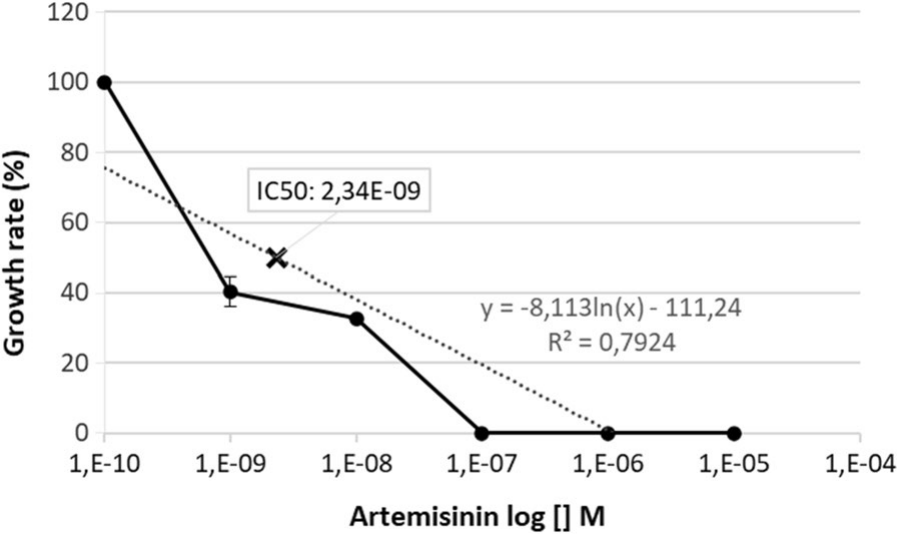
*P. falciparum* 3D7 parasite growth rate after 48 h of incubation with various concentration of artemisinin. The IC_50_ of artemisinin was detected at 2.34 nM

*Plasmodium falciparum* parasite growth in various concentrations of ATA is shown in Table 1. Cultivation of the parasite in the presence of a wide concentration range of ATA exhibited a dose-dependent pattern with a weak growth inhibition at the lower concentrations (0.1 nM–10^–1^ mM) but marked inhibition at higher doses 100 µM (Fig. 1). No obvious effect of the inhibitors on the amount of RBC observed. Using a probit analysis, the IC_50_ of ATA was found at the 634 µM. In comparison, the IC_50_ of artemisinin, which was used as control positive compound, was 2.34 nM (Fig. 2).

**Table 1:** Effect of PMCA4 inhibition on parasite count at 48 h after initiation of culture

Drug concentration (M)	Parasite count at 48 h, %					
	Artemisinin		ATA		Resveratrol	
	(1*)	(2)	(1)	(2)	(1)	(2)
10^–10^ (Untreated)	6.4	4.7	4.2	3.1	9	17
10^–9^	2.3	2.1	–	–	15	8
10^–8^	2.1	1.6	–	–	12	11
10^–7^	0	0	3.3	2.8	1.8	1.2
10^–6^	0	0	3.9	2.7	0.7	1.3
10^–5^	0	0	3.4	3.3	0.7	1.3
10^–4^	0	0	0.8	1	1.4	1.6
10^–3^	0	0	0	0	0.9	0.8

^*^ = assay 1

#### Anti-malarial activity of PMCA4 inhibitor, resveratrol, in vitro

**Fig. 3:**
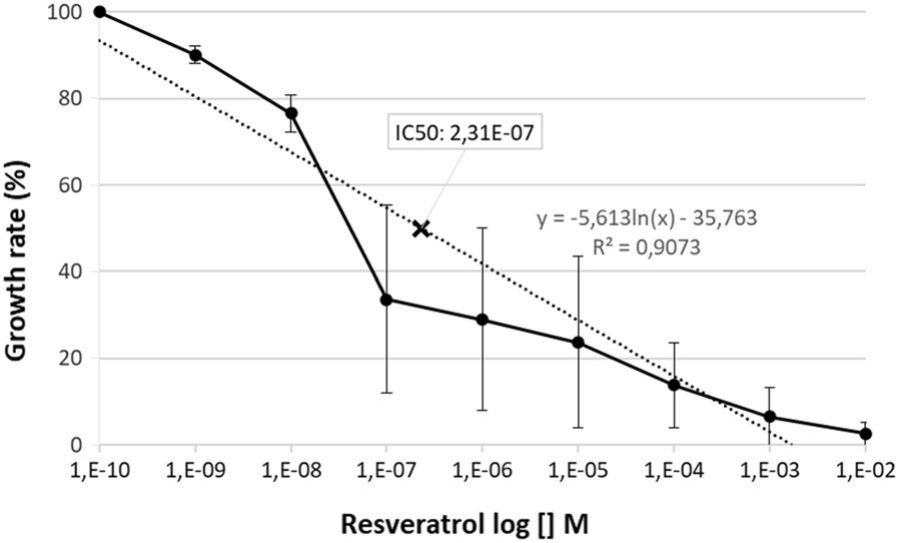
*P. falciparum* 3D7 parasite growth rate after 48 h of incubation with various concentration of resveratrol. The IC_50_ was detected at 0.231 µM

The parasitaemia of the parasite culture in the presence of various concentrations of resveratrol is shown in Table 1. Cultivation of *P. falciparum* in the presence of resveratrol at the concentration ranges of 1 nM–1 mM exhibited a dose-dependent growth inhibition pattern with IC_50_ at 0.231 µM (Fig. 3). Compared to ATA, resveratrol showed stronger inhibition against *P. falciparum* growth in vitro.

### Effect PMCA4 inhibitor Aurintricarboxyclic Acid (ATA) in vivo against *Plasmodium berghei* in experimental cerebral malaria (ECM) model

**Fig. 4:**
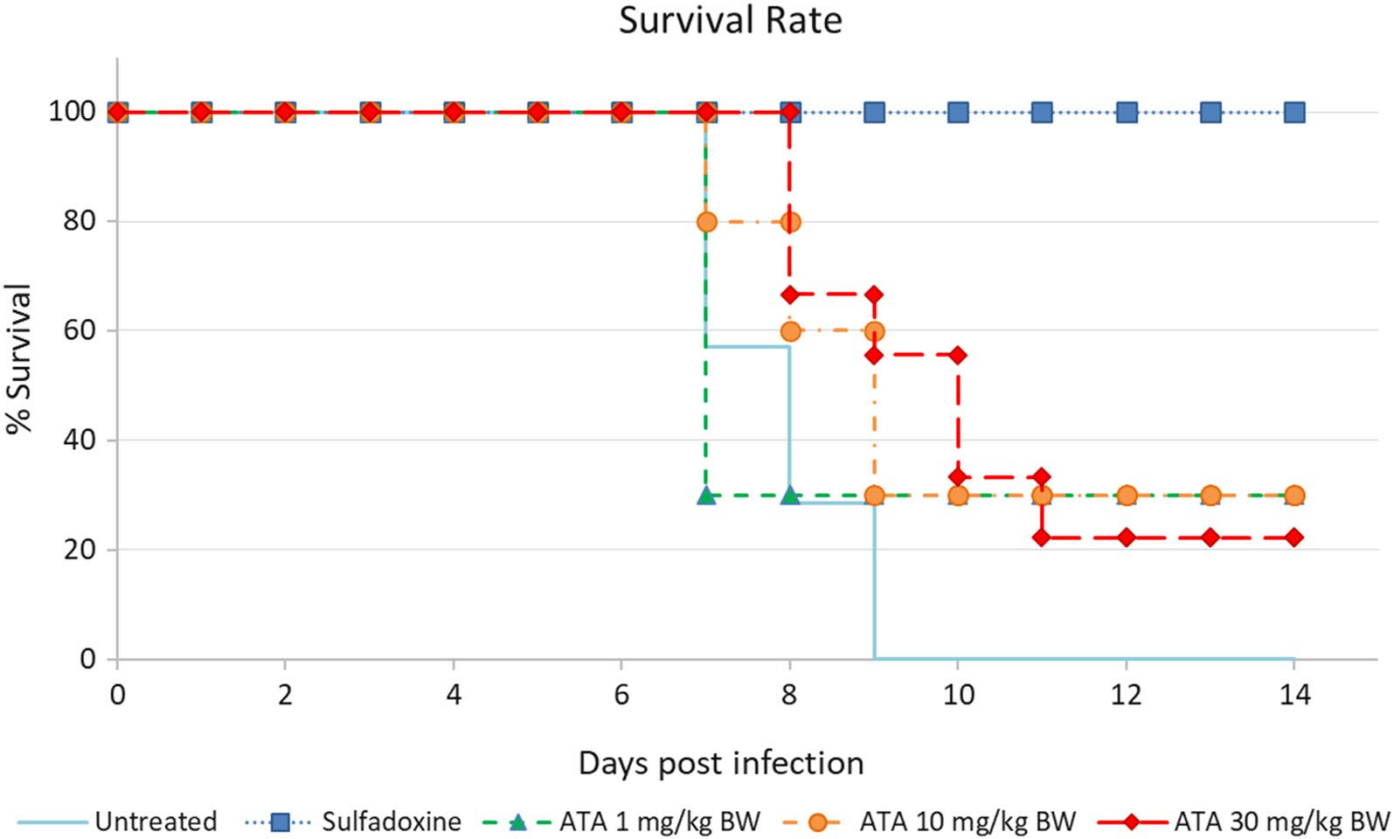
The effect of 30 mg/kg BW ATA on survival rate of experimental cerebral malaria model mouse infected with *P. berghei* ANKA treatment with 30 mg/kb BW. ATA showed longer survival rate whereas the other concentrations did not significantly differ than the control untreated group

All mice in the untreated group succumbed to CM on day 7–9 post infection whilst all mice in the sulfadoxine group survived until the end of the experiment. Treatment with various concentrations of ATA yielded similar survival rates with the untreated groups except for the highest dose (30 mg/kg BW) that showed a significantly higher survival rate (Fig. 4). When compared to sulfadoxine, however, all ATA-treated groups including that with the highest dose had significantly lower survival rates. These suggest that ATA 30 mg/kg BW may increase survival after infection with *P. berghei,* but was not as effective as sulfadoxine.

Analysis on the body weight changes and the red blood cells concentrations on the untreated and ATA-treated groups revealed a non-significant pattern in which both groups exhibit a decrease in bodyweight and red blood cell concentrations during the course of the experiment. Similar pattern was also found on the degree of parasitaemia in treated and untreated groups before the mice succumbed (Table 2).

**Table 2:** Effect of PMCA4 inhibition of ATA on the body weight changes and the red blood cells concentrations

Dosage	Day post infection	Monitoring indicators				
		Body weight		RBC		
		Weight (g)	Loss (%)	Count	Concentrations	Loss (%)
Sulfadoxine 25 mg/kg BW	1	20.75 (± .50)	0	89 (± 12.25)	4.5E+09 (± 6.1E+08)	0
	9	21.25 (± 1.71)	2.41	78 (± 14.31)	3.9E+09 (± 7.2E+08)	− 12.36
	14	21.75 (± 1.26)	4.82	101.5 (± 19.28)	5.1E+09 (± 9.6E+08)	14.04
Untreated	1	20.2 (± 1.48)	0	73.4 (± 10.31)	3.7E + 09 (± 5.2E+08)	0
	9	21.2 (± .84)	4.95	45 (± 12.23)	2.3E+09 (± 6.1E+08)	− 38.69
	14	18.4 (± .55)	− 8.91	32 (± 9.08)	1.6E+09 (± 4.5E+08)	− 56.40
ATA 30 mg/kg BW	1	17.00 (± 1.83)	0	69.7 (± 11.67)	3.5E+09 (± 5.8E+08)	0
	9	16.57 (± .53)	− 2.52	61 (± 13.60)	3.1E+09 (± 6.8E+08)	− 12.50
	14	16.17 (± .98)	− 4.90	36.3 (± 17.04)	1.8E+09 (± 8.5E+08)	− 47.95

### Anti-malarial activity of PMCA4 inhibitor, resveratrol, in vivo

**Fig. 5:**
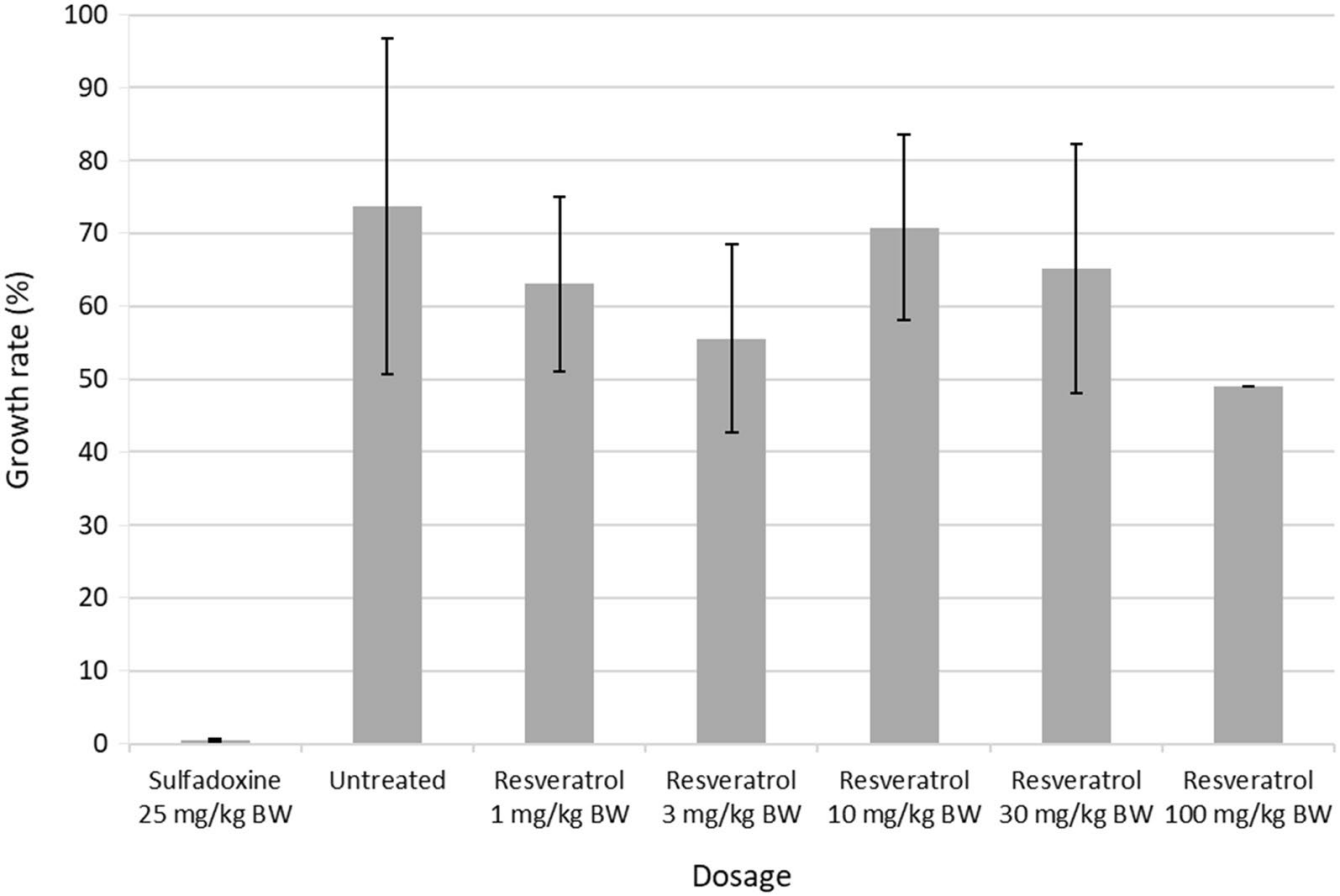
Histogram of the *Plasmodium yoelii* growth rate in BALB/c mice in the presence or absence of PMCA inhibitor, resveratrol. Sulfadoxine is used as positive control

Treatment of the *P. yoelii*-infected mice with intraperitoneal resveratrol at the concentrations of 1 mg-100 mg/kg BW revealed a non-significant inhibition of the growth rate at day 4, except at the concentration of 100 mg/kg BW where a 37% growth inhibition was shown (Fig. 5).

## Discussion

The identification of PMCA4 SNPs as significant genetic determinants for malaria severity has prompted the idea that PMCA4 may be a novel target for malaria treatment. PMCA4 is coded for by the *Atp2b4* gene located on chromosome 1 (Iq32) and several single nucleotide polymorphisms (SNPs), namely rs10900585; rs1541254; rs1541255; and rs4951074, have been implicated in susceptibility to severe malaria in genome wide association [4] and epidemiologic studies in Africa and Oceania [19, 20]. The molecular basis for the association of PMCA4 and severe malarial disease is currently still unclear but owing to the position of the SNPs in the promoter region of the gene, the effect might be mediated by the altered expression of the PMCA4, as shown by Zambo et al. [21]. Indeed, an expressed quantitative trait loci (eQTL) analysis has shown that PMCA4 SNPs are correlated with the level of PMCA4 expression in RBCs [8].

The findings of this study show that ATA and Resveratrol exhibited a potent anti-malarial activity on *P. falciparum* growth in the in vitro RBC cultures. The anti-malarial properties of the two PMCA4 inhibitors may be linked to the role of this protein in RBCs [14]. Inhibition of PMCA4 results in excess intracellular calcium which then activates a calcium-activated potassium channel (the Gardos channel), resulting in potassium efflux, RBC volume loss, and elevated mean corpuscular haemoglobin concentration (MCHC). Hydration of RBC has been linked with clinical severity of blood-stage malaria in the haemoglobin disorder sickle cell disease [22], and with infectivity of *P. falciparum* merozoites into RBCs [23].

At the in vivo experiment, particularly to determine the effect of ATA on cerebral malaria, the effect on the mice survival was noted at higher dose. Similar pattern was also observed with resveratrol on the parasite growth. The findings are in accordance with previous finding whereby ablation of PMCA4 confer a slight protection against cerebral malaria and did not significantly alter peripheral parasite burdens [8, 23].

Aurintricarboxylic acid (ATA) was identified as a potent PMCA4 inhibitor previously with IC_50_ at 150 nM at cardiac fibroblast [24]. ATA also inhibits other enzymes, such as nucleases, calpain and influenza virus neuraminidase at higher concentrations [25–28]. This study found that the anti-malarial property of ATA in vitro was relatively potent with IC_50_ at 634 mM, but in in vivo experiment, particularly to determine its activity against cerebral malaria, its activity was modest and was only observed at higher dose. Therefore, it is suggested that either the anti-malarial property of ATA in vivo is not mediated through the PMCA4 inhibition or their different pharmacokinetic property of the compound when given intraperitoneally. For the ATA activity in vivo, delivery through oral instead of intraperitoneal injection may be considered.

Likewise, resveratrol which exhibits a potent anti-malarial property in vitro has currently been available at the market as antioxidant formula indicated for a wide range of diseases. In addition to its property as pan PMCA inhibitor, the compound also demonstrated efficacy on various diseases in different dosage [29]. The resveratrol PMCA inhibition property was attained at micromolar range in various cells [13, 30]. Nevertheless, this anti-malarial property was observed at higher dose (100 mg/kg BW) through the intraperitoneal prescription in vivo using dimethyl sulfoxide as solvent. Therefore, effort to increase its solubility in water as well as the possibility for oral prescription might be worthy to pursue. Overall, it is suggested that the anti-malarial property of resveratrol is mediated through its property as PMCA4 inhibitor. As to the ATA modest effect in vivo, this phenomenon may relate to the pharmacokinetic property of the compound when given intraperitoneally or may also indicate other unspecific targets as reported before [15, 31]. The intrinsic antioxidant capacity of the resveratrol molecule and its ability to trigger the activation/repression of a wide range of membrane receptors, kinases and other enzymes have turned the quest for a molecular mechanism of action into an epic task [31]. To our knowledge, this is the first report of the anti-malarial activity of resveratrol and certainly deserves further exploration to circumvent the everlasting problem of anti-malarial drug resistance.

In conclusion, we have explored the anti-malarial activities of two compounds that are known as PMCA4 inhibitors. The results indicated that PMCA4 represents a promising novel target for anti-malarial development. Further study to determine biochemical pathways affected by the PMCA4 inhibitors to exert its anti-malarial property and drug delivery is currently on going.

## Data Availability

All relevant data are within the manuscript.
